# A Robust Framework Fusing Visual SLAM and 3D Gaussian Splatting with a Coarse-Fine Method for Dynamic Region Segmentation

**DOI:** 10.3390/s25175539

**Published:** 2025-09-05

**Authors:** Zhian Chen, Yaqi Hu, Yong Liu

**Affiliations:** 1 Department of Computer Science and Engineering, Southern University of Science and Technology, Shenzhen 518000, China; 12211829@mail.sustech.edu.cn; 2 School of Computer Science and Engineering, Nanjing University of Science and Technology, Nanjing 210094, China; augusthyq@163.com

**Keywords:** visual slam, coarse-fine method, dynamic region segmentation, 3D gaussian splatting

## Abstract

Existing visual SLAM systems with neural representations excel in static scenes but fail in dynamic environments where moving objects degrade performance. To address this, we propose a robust dynamic SLAM framework combining classic geometric features for localization with learned photometric features for dense mapping. Our method first tracks objects using instance segmentation and a Kalman filter. We then introduce a cascaded, coarse-to-fine strategy for efficient motion analysis: a lightweight sparse optical flow method performs a coarse screening, while a fine-grained dense optical flow clustering is selectively invoked for ambiguous targets. By filtering features on dynamic regions, our system drastically improves camera pose estimation, reducing Absolute Trajectory Error by up to 95% on dynamic TUM RGB-D sequences compared to ORB-SLAM3, and generates clean dense maps. The 3D Gaussian Splatting backend, optimized with a Gaussian pyramid strategy, ensures high-quality reconstruction. Validations on diverse datasets confirm our system’s robustness, achieving accurate localization and high-fidelity mapping in dynamic scenarios while reducing motion analysis computation by 91.7% over a dense-only approach.

## 1. Introduction

Simultaneous Localization and Mapping (SLAM) represents a pivotal research direction in the field of mobile robotics, enabling robots to perceive their surroundings and navigate accurately. This technology has found widespread application in diverse fields, including augmented reality, autonomous driving, and virtual reality. Visual SLAM leverages images as the primary means of environmental perception. Its notable advantages—low cost, light weight, and small size—have attracted significant attention from the SLAM research community.

The visual SLAM algorithms, which are typically categorized into the feature-based method and the direct method, are extensively utilized across various applications. Specifically, the feature-based visual odometry system [1,2] excels in textured scenes by extracting and matching image feature points to achieve motion tracking and map construction. However, traditional visual SLAM systems inherently rely on the assumption of a static environment, posing significant challenges for achieving accurate localization and mapping in dynamic environments. To address these challenges and enhance the accuracy of SLAM systems in dynamic scenarios, several tailored solutions have been proposed in recent SLAM algorithms [3,4]. These solutions incorporate diverse methodologies, such as leveraging semantic information for dynamic prediction and object detection. The primary objective of these methods is to mitigate the impact of dynamic objects and thereby substantially improve localization accuracy. Nevertheless, methods that solely rely on object detection or semantic priors may inadvertently filter out static feature points within dynamic objects, resulting in a substantial reduction in the number of available feature points and a decline in localization accuracy of the system, particularly in environments with numerous dynamic objects.

In addition, the evolution of SLAM from traditional methods, which primarily focused on enhancing localization accuracy, to neural radiance fields (NeRFs) that provide rich scene representations has increasingly highlighted the significance of SLAM in map representation. This development has played a crucial role in downstream tasks. Visual SLAM has introduced a variety of map representation methods, such as point clouds, meshes, and voxels. Recently, NeRF-based novel view synthesis [5] has gained popularity due to its high-quality 3D reconstruction capabilities, driving the advancement of numerous dense neural SLAM methods. However, volume rendering requires considerable computation during NeRF optimization, leading to poor real-time performance and an inability to render full-resolution images. Three-dimensional Gaussian Splatting (3D GS) [6],a rapidly evolving novel image rendering technique, achieves high-quality full-pixel novel view synthesis and real-time scene rendering within a GPU-accelerated framework. By leveraging 3D Gaussians as a flexible and efficient representation and combining the advantages of both discrete and continuous representation methods, 3D GS not only surpasses traditional methods in terms of noise reduction and rendering quality but also significantly boosts rendering speed. The integration of 3D GS and SLAM represents a current research direction [7,8,9], where the incorporation of a 3D GS renderer for image rendering can reduce the cost of view reconstruction.

**Figure 1:**
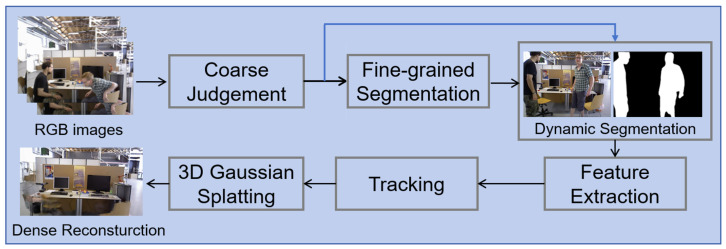
Brief system overview.

To tackle the shortcomings in localization and mapping prevalent in existing SLAM methods, we developed a framework incorporating visual SLAM and 3D Gaussian Splatting (as shown in Figure 1). This framework introduces a combined coarse-fine method for dynamic region segmentation, marking a significant advancement in camera pose refinement and the construction of dense, accurate maps tailored for dynamic visual SLAM scenarios. The key contributions of this work are summarized as follows:

1. We propose a cascaded coarse-fine approach for dynamic region segmentation. It first leverages contour-based sparse optical flow for an efficient coarse motion check. For complex, non-rigid targets with partial or localized motion, it then applies a dense optical flow clustering within the bounding box for local, fine-grained motion segmentation. This integration significantly enhances the efficiency, accuracy, and robustness of dynamic target segmentation, contributing to improved camera pose estimation and map construction in dynamic environments.
2. By combining visual odometry with the 3D Gaussian Splatting framework, our system maintains a hybrid map, incorporating feature points, rotations, scales, densities, and spherical harmonic coefficients. On the one hand, we optimize tracking performance using a factor graph solver. On the other hand, we utilize backpropagation to minimize the loss between original and rendered images, enabling rapid and high-quality dense map construction.

## 2. Related Work

### 2.1. Visual SLAM Systems for Dynamic Environments

Most real-world environments are dynamic, with moving objects such as pedestrians, vehicles, animals, and so forth. Traditional SLAM algorithms are typically based on the assumption of static environments. The presence of dynamic objects leads to changes in scene geometry, affecting the accuracy of system pose estimation, and causing localization drift or failure. Furthermore, dynamic objects can produce artifacts in the map, which not only impact the map’s accuracy but also hinder the execution of downstream tasks such as path planning and environment perception. Therefore, considerable research efforts have been dedicated to detecting and filtering out dynamic objects, excluding the interference of dynamic features, to enhance the accuracy of SLAM in dynamic environments.

Currently, the methods employed by SLAM systems to handle dynamic objects in scenes are mainly divided into two categories. (1) Methods based on multi-view geometry. Alcantarilla [10] calculates the camera pose using consecutive frame images and based on this initially estimates the dense 3D optical flow of the images. Subsequently, by computing the Mahalanobis distance of matching points between two frames and comparing it with a preset threshold, he effectively eliminates unqualified outliers, achieving the identification and removal of dynamic objects. Tan [11] adopts the RANSAC algorithm to compute the transformation matrix between adjacent frames and applies this matrix to the previous frame image to generate a transformed image through matrix operations. Then, the transformed image is subtracted from the current frame image, combined with subsequent processing steps, ultimately obtaining segmentation results and realizing the identification and separation of dynamic objects. (2) Methods based on semantic priors. Sheng [12] utilizes Mask RCNN for image semantic segmentation to identify prior dynamic regions in the environment. Pixels outside these dynamic regions and their neighboring pixels are considered static and are directly integrated into the DSO system, achieving significant results on the TUM dataset. Bescos [4] combines the Mask R-CNN semantic segmentation network with multi-view geometry algorithms to accurately detect and remove dynamic objects and proposes the DynaSLAM system based on the ORB-SLAM2 framework. Zhong [13] employs the SSD network to label dynamic objects in the scene based on prior information, thereby filtering out dynamic feature points and using only the remaining static points for map construction. RDS-SLAM [14], building upon the ORB-SLAM3 system, introduces an independent semantic segmentation thread that leverages mobility probabilities to transmit semantic information to the tracking thread. This design enhances the system’s real-time performance while effectively reducing interference from dynamic objects.

**Figure 2:**
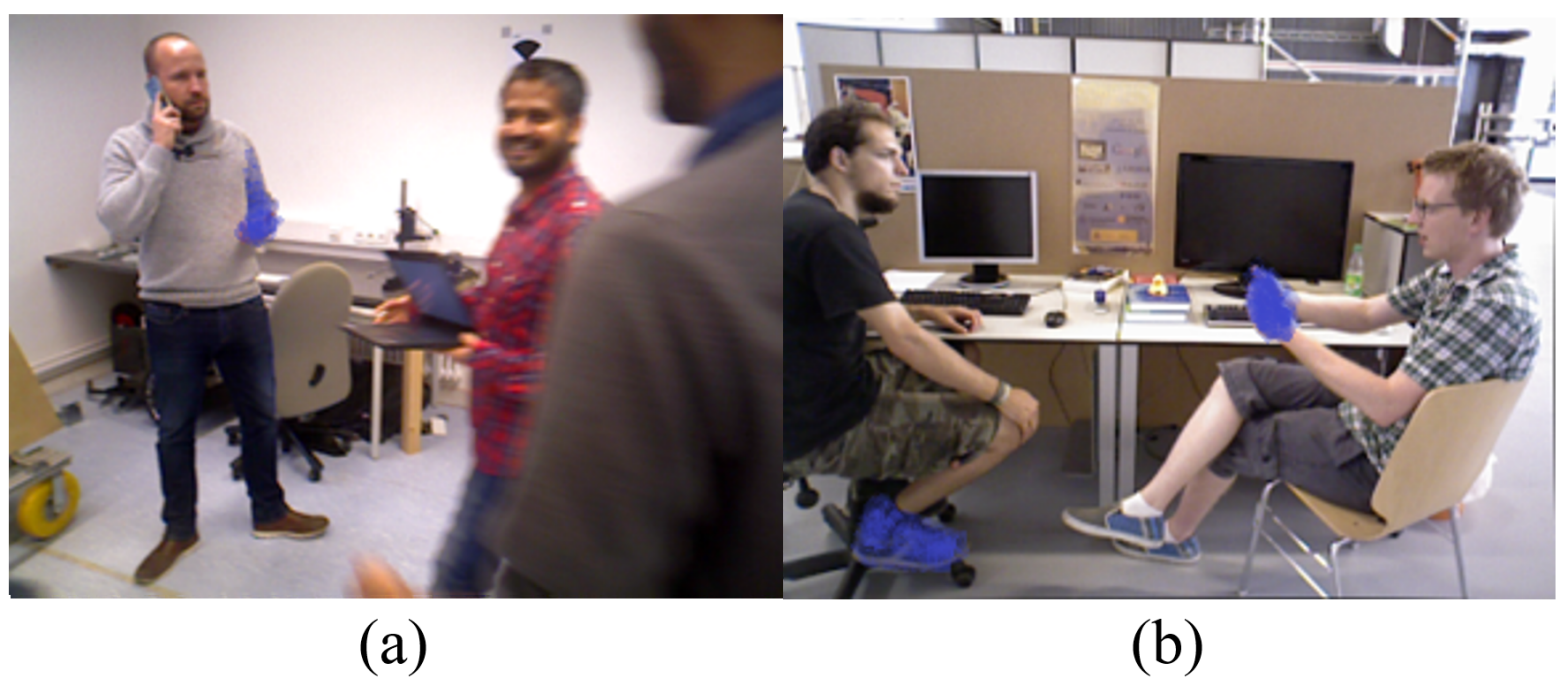
Scenarios where issues arise in dynamic visual SLAM. (**a**) A scene with multiple actors, including one subject making a call (local hand motion) and others causing motion blur. (**b**) A scene depicting subtle limb movements from two seated individuals in conversation. Both scenarios present challenges of non-rigid, local motion for traditional segmentation methods.

Despite demonstrating certain processing capabilities in most dynamic scenes, methods such as multi-view geometry exhibit relatively low robustness. Methods relying on prior semantic information, however, are limited by the types and quantities of dynamic objects in the training set. When dynamic object types not covered in the training set appear in the scene, or when prior dynamic objects occupy a significant proportion (as shown in Figure 2), the number of available feature points drops dramatically, potentially leading to impaired system performance or even failure. To address these challenges, many studies have explored methods based on optical flow and scene flow for segmenting dynamic regions. DS-SLAM [3] combines the SegNet network with motion consistency detection technology to successfully eliminate dynamic feature points in dynamic scenes. STDyn-SLAM [15] integrates optical flow, SegNet, and depth maps to comprehensively detect dynamic objects, using the principle of motion consistency to identify dynamic points. DytanVO [16] utilizes the rigid motion segmentation network RigidMask [17] to extract dynamic optical flow, achieving joint optimization of motion segmentation and pose estimation. SG-SLAM [18] also combines object detection and sparse optical flow techniques, relying on the principle of motion consistency to identify dynamic feature points. These methods exhibit significant potential in enhancing the processing capabilities for dynamic scenes.

More recently, with the rise of neural SLAM, research has shifted towards modeling dynamic elements directly within the neural representation itself. Instead of simply masking out dynamic features, these state-of-the-art approaches often decompose the scene into static and dynamic components. The foundational idea, demonstrated in works like D-NeRF [19], involves learning a neural deformation field to map a dynamic scene to a canonical, static representation. This concept has been extended to recent 3D Gaussian Splatting frameworks, where systems like Dynamic 3D Gaussians [20] learn separate sets of Gaussians for the static background and dynamic foreground elements, achieving real-time, high-fidelity reconstruction of moving objects from a single camera. However, these neural dynamic SLAM systems, while powerful, often come with a significant computational burden. They typically require training multiple complex neural networks or deformation fields simultaneously and can struggle to converge robustly in all scenarios, making them less suitable for many robotics applications.

Currently, most methods based on optical flow and semantic information focus on the overall segmentation of dynamic objects, with deficiencies in fine-grained motion detection. In practical applications, human behavior encompasses not only overall displacement but also subtle movements of local body parts such as hands and heads. These fine actions are relatively small in amplitude and occupy a minor proportion of the entire human body, making them prone to being overlooked during global judgment. Consequently, they cannot be effectively detected and incorporated into motion analysis (as shown in Figure 2). When a significant amount of such undetected local motion exists, it can severely compromise system accuracy. This creates a critical challenge for dynamic SLAM systems. On one hand, methods that aggressively segment entire object categories risk inadvertently filtering out static feature points within those objects, substantially reducing feature density and degrading performance in dynamic-cluttered scenes. On the other hand, methods that fail to detect these subtle local motions will erroneously treat dynamic points as static, corrupting camera pose estimation and the map itself. While employing dense optical flow across all potential moving objects could provide the necessary detail for fine-grained segmentation, its prohibitive computational cost makes it unsuitable for real-time SLAM applications. However, we observe that the motion of sparse feature points tracked along an object’s contour often provides a strong-yet-efficient cue to its dominant motion. A significant discrepancy between the contour’s motion and the background can reliably indicate global movement, while consistency suggests a static or complex internal motion scenario.

### 2.2. Dense Mapping in SLAM: From NeRF to 3D Gaussian Splatting

In parallel to advancements in dynamic SLAM, there has been a significant evolution in dense 3D scene representation for mapping. Traditional explicit methods like point clouds or meshes often struggle with storage efficiency and high-resolution details. This led to the rise of implicit representations using neural networks. Neural Radiance Fields (NeRFs) [5] became a landmark, enabling high-quality novel view synthesis. Subsequent works improved upon NeRF limitations, such as slow rendering speeds with Instant-NGP [21], aliasing artifacts with Mip-NeRF [22], point-based acceleration with Point-NeRF [23], and large-scale capabilities with Mega-NeRF [24] and Block-NeRF [25].

The integration of NeRF into SLAM, seen in systems like iMAP [26], NICE-SLAM [27], Point-SLAM [28], and Orbeez-SLAM [29], promised photorealistic, dense map reconstruction. However, NeRF-based SLAM systems are often hampered by high computational complexity, which conflicts with the real-time requirements of robotics.

A pivotal breakthrough came with 3D Gaussian Splatting (3DGS) [6], which offers a highly efficient explicit representation that matches or exceeds NeRF’s rendering quality while achieving real-time performance. This has spurred a new wave of SLAM research. Systems like GS-SLAM [8], Gaussian Splatting SLAM [30], and Photo-SLAM [9] have successfully combined traditional SLAM front-ends with 3DGS-based mapping back-ends. Nevertheless, a critical limitation persists: none of these pioneering 3DGS-SLAM systems are designed to effectively handle dynamic environments. The presence of moving objects leads to severe artifacts in the reconstructed map and a significant degradation in localization accuracy. This work aims to bridge this gap by introducing a robust dynamic object handling mechanism into a state-of-the-art 3DGS-SLAM framework.

## 3. System Description and Methods

**Figure 3:**
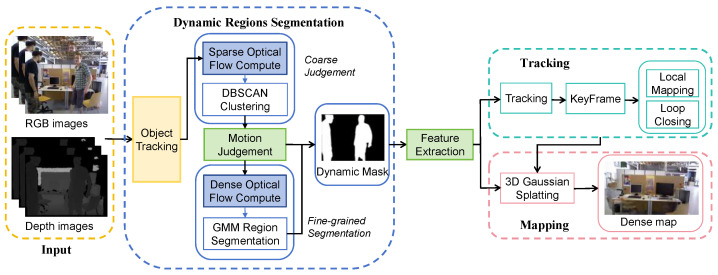
System overview. Our system consists of three main components: dynamic region segmentation, tracking, and mapping. We effectively filter out dynamic feature points through the proposed coarse-fine dynamic region segmentation module. The tracking module then proceeds to estimate camera pose and construct a sparse geometric map, utilizing only the filtered static feature points. Simultaneously, the mapping module employs 3D Gaussian Splatting rendering based on keyframes and static map points to construct a dense map.

The system framework, as depicted in Figure 3, builds upon ORB-SLAM3 as its foundational structure. Based on the proposed coarse-fine dynamic region segmentation module, our system filters out dynamic feature points. The filtered static feature points serve dual functions within the whole system. On one hand, they are utilized for tracking, enabling the system to optimize camera pose estimation. On the other hand, these static map points facilitate 3D Gaussian Splatting rendering, effectively mitigating the impact of dynamic objects and enabling superior map construction. By focusing exclusively on static feature points, the system ensures both accurate tracking and high-quality mapping of the static environment.

### 3.1. System Overview

Our system comprises three main components: dynamic region segmentation, tracking, and mapping.

During the preprocessing stage, we apply DeepLab2 [31] to segment the scene and identify movable objects. Then, the RGB images, depth images, and corresponding instance masks are input into the system.

Based on the instance segmentation results, we achieve a multi-target tracking process similar to ByteTrack [32]. We identify bounding boxes for potential moving objects and employ Kalman filters to predict their positions in the next frame. The Hungarian algorithm is employed to associate and match the detected bounding boxes with the predicted ones, with Intersection over Union (IOU) and cosine similarity serving as similarity metrics for the matches. We create a Kalman tracker for each detected object, enabling the system to be applied in scenarios with multiple potential moving targets.

For each successfully tracked potential moving object, we employ a hierarchical, coarse-to-fine strategy for motion analysis. This process involves two main stages: (1) Coarse Motion Analysis: We first perform an efficient check using sparse optical flow. Features are extracted in annular regions along the object’s contour, and their motion is analyzed via DBSCAN clustering to quickly classify targets as either clearly static or globally dynamic. (2) Fine-grained Motion Segmentation: For ambiguous objects that pass the initial screening (typically non-rigid bodies like humans), we then compute a dense optical flow field. Gaussian Mixture Models (GMMs) are subsequently used to cluster these dense flow features, enabling fine-grained segmentation of locally moving regions. By integrating coarse judgments and fine-grained segmentation, we can more accurately obtain masks for dynamic regions.

The tracking module subsequently undertakes the task of estimating camera pose and constructing a sparse geometric map. This process utilizes only the filtered static feature points, which have been selected to exclude dynamic elements that could compromise the accuracy of the localization. Based on the camera pose estimates and the static feature points, the module constructs a sparse geometric map of the environment.

Meanwhile, our system adopts a hybrid map representation that combines geometric features with 3D Gaussian. On the one hand, it fully leverages the precise geometric information provided by feature map points to achieve rapid and accurate pose estimation. On the other hand, we designed a 3D Gaussian optimization strategy based on a Gaussian pyramid and a 3D Gaussian densification algorithm based on geometric features to refine the 3D Gaussian parameters. By extracting static feature points using the dynamic component segmentation module, the system can mitigate the impact of dynamic objects during the rendering process. This enables the creation of detailed static background maps, even in environments characterized by significant dynamic activity.

### 3.2. Dynamic Region Segmentation

The dynamic region segmentation module is crucial for the system to operate effectively in dynamic environments. Based on the results of instance segmentation and object tracking, we obtain matched object bounding boxes between consecutive frames. For each potential moving object, instead of directly applying computationally expensive methods, we propose a cascaded analysis pipeline that leverages both sparse and dense optical flow.

Sparse Optical Flow for Coarse Motion Analysis. As the first stage, we employ a lightweight, sparse optical flow method for an efficient coarse motion assessment. This approach, based on the Lucas–Kanade (LK) algorithm, tracks a limited set of high-quality feature points (corners) within annular regions surrounding the object’s contour. By clustering the resulting sparse motion vectors using DBSCAN and comparing the dominant motion against the background, we can rapidly filter out objects that are clearly static or undergoing simple, global translation. This pre-screening step significantly reduces the computational load by avoiding unnecessary dense analysis on every object.

Dense Optical flow for Fine-grained Motion Segmentation. For objects flagged as ambiguous by the sparse-flow stage (e.g., non-rigid bodies with complex internal movements), we then proceed to a more detailed analysis using dense optical flow. Optical flow represents the instantaneous velocity of pixel movements between two frames, leveraging changes in pixel intensity to establish dense correspondences. This method generally relies on three fundamental assumptions: (1) brightness constancy, (2) small motion, and (3) spatial consistency. To precisely capture fine-grained local motion, this paper employs the RAFT (Recurrent All-Pairs Field Transforms) algorithm [33] for dense optical flow computation. Through an iterative optimization process, RAFT accurately estimates a dense field of optical flow vectors for each pixel within the object’s bounding box, providing a solid foundation for our subsequent segmentation modules.

The complete dynamic region segmentation module is thus divided into two main parts based on the outputs of our cascaded flow analysis: coarse object judgement and fine-grained motion segmentation.

#### 3.2.1. Coarse Motion Judgement

Our cascaded analysis pipeline begins with an efficient global motion assessment, outlined in Algorithm 1, to rapidly identify objects that are either static or undergoing simple, uniform motion. Instead of the costly dense optical flow, this stage leverages a robust sparse optical flow approach combined with motion clustering.

**Figure 4:**
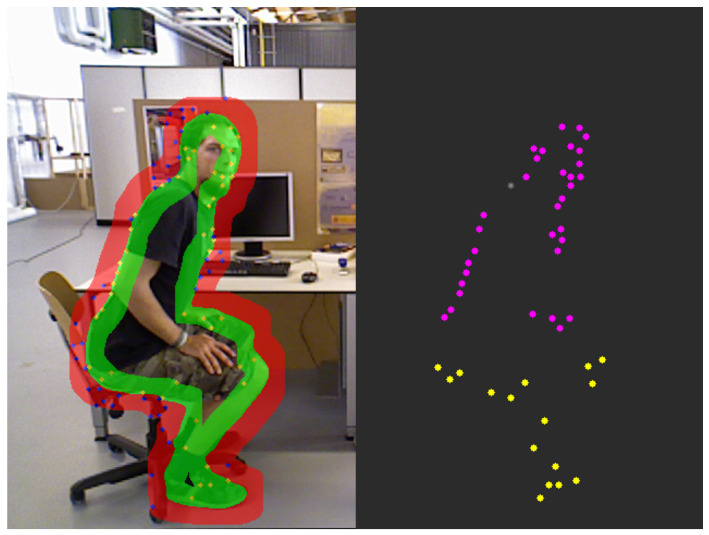
Annular masks for sparse feature extraction. The outer ring (red) captures background motion, while the inner ring (green) captures the object’s peripheral motion. Feature points (yellow/blue) on the left picture are extracted only within these rings.

First, for each tracked object, we generate annular masks representing regions immediately inside and outside its contour, as depicted in the left picture of Figure 4. This is achieved through morphological dilation and erosion on the object’s instance mask. These annular regions serve as precise masks for extracting a sparse set of high-quality feature points using the goodFeaturesToTrack algorithm. To enhance robustness, particularly for smaller or less-textured objects like chairs, we dynamically adjust the feature extraction parameters based on the object’s class.

Subsequently, to track these sparse feature points between consecutive frames, we employ the GPU-accelerated Lucas–Kanade (LK) pyramidal optical flow algorithm (cv::cuda::SparsePyrLKOpticalFlow). For our implementation, we configure it with a 21 × 21 pixel search window, 3 pyramid levels, and a maximum of 30 iterations per level, a setup which ensures a strong balance between tracking accuracy and real-time performance. The resulting motion vectors from the inner ring are then fed into the DBSCAN (Density-Based Spatial Clustering of Applications with Noise) algorithm. Using a neighborhood distance eps of 0.2 and requiring a minimum of 5 points (min_samples), DBSCAN groups features into distinct motion clusters based on a composite distance metric that weights spatial proximity ($w_{\mathrm{space}}=0.3$) and flow vector similarity ($w_{\mathrm{flow}}=1.2$). This allows the algorithm to automatically identify the dominant motion cluster within the object, effectively filtering out noise and minor local movements.

The final decision is made through a hierarchical voting process. First, we identify the largest motion cluster from DBSCAN as the object’s dominant motion. If this cluster’s size relative to the total number of tracked points is below a threshold $\tau_{ratio}$, which we set to 0.5, the object’s motion is deemed chaotic and immediately flagged for dense analysis. This value ensures that a "dominant" motion is shared by at least a majority of points; a lower value would risk misinterpreting noisy motion as coherent, while a higher value is oo strict for non-rigid objects with complex movements. Otherwise, the representative motion of this dominant cluster, calculated using the median of its flow vectors for robustness against outliers, is compared against the median motion of the background points from the outer ring. If the difference in magnitude or angle exceeds predefined motion thresholds, $\tau_{\mathrm{motion}}$, the object is classified as globally dynamic. These thresholds (e.g., 2.0 pixels in magnitude, 15 degrees in angle) are critical for distinguishing true object movement from apparent motion caused by camera ego-motion. Stricter values could cause false positives on static objects due to minor noise, whereas more lenient values might fail to detect subtle but independent object movements. If the dominant motion is consistent with the background, we then check the ratio of non-dominant points (other clusters and noise). If this outlier ratio is significant, determined by a threshold $\tau_{\mathrm{outlier}}$ set to 0.15, particularly for non-rigid objects like a ’person’, the object is flagged for a more detailed local motion analysis using dense optical flow. This value is tuned to capture meaningful local dynamics, such as a waving arm, without being overly sensitive to minor tracking noise. A lower value triggers the dense analysis too frequently, while a higher one can miss important non-rigid movements.This cascaded approach ensures that the computationally expensive dense flow is only invoked when absolutely necessary. This methodology is inherently more robust than methods that rely on a single, averaged motion vector. By segmenting motion into clusters, our system can identify the true dominant motion without being skewed by a minority of outlier points or localized movements, thus avoiding the common pitfalls of mean-based analysis.

**Figure 5:**
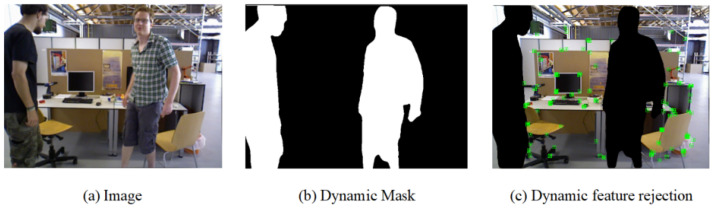
Dynamic mask results obtained through overall dynamic target judgement. (**a**) The original image, (**b**) the dynamic mask, and (**c**) the feature extraction effect on static regions.

The right panel of Figure 4 visualizes the clustering results from DBSCAN, effectively illustrating the capability of our motion segmentation approach. In this example, the feature points within the inner ring of the object are successfully partitioned into two dominant motion clusters: an upper body cluster exhibiting motion (represented by magenta points) and a static lower body cluster (yellow points). This segmentation accurately captures the semantics of the action—a person bending over. Since the ratio of static points is significant, the system correctly identifies this as a case of complex, non-rigid motion, where only local parts of the object are moving. Consequently, it flags the object for a fine-grained analysis using dense optical flow rather than misclassifying it as a simple global movement. Based on the aforementioned process, we are able to successfully identify targets that exhibit significant motion amplitudes. Figure 5 provides a clear visualization of the region segmentation results for the global judgement of dynamic objects within the image.

#### 3.2.2. Fine-Grained Motion Segmentation

Our coarse-grained motion analysis, based on sparse optical flow and DBSCAN clustering, excels at efficiently identifying objects that are either clearly static or undergoing simple, global motion. However, this holistic judgment is insufficient for complex scenarios, such as a non-rigid body exhibiting localized movements (e.g., a seated person waving an arm). In this case, the dominant motion cluster (the torso) remains static relative to the background, while a significant secondary motion cluster (the arm) exists. Our coarse stage is designed to specifically detect this ambiguity. When an object, particularly a ’person’, is found to have a dominant static cluster but also a notable ratio of outlier or secondary motion points (as determined by the logic in Algorithm 1, returning a NON_RIGID state), the framework transitions to a fine-grained analysis to resolve the uncertainty.
**Algorithm 1** Robust Coarse Judgement via Sparse Flow and Clustering**Input:** Current image $I_{t}$, Previous image $I_{t-1}$, Object instance mask $mask_{t}$, Tracked object obj**Output:** Motion state $S_{obj}\in \left\{STATIC,RIGID\_MOTION,NON\_RIGID\right\}$  1:$contour\leftarrow \mathrm{findContours}(mask_{t})$  2:$mask_{inner},mask_{outer}\leftarrow \mathrm{createAnnularMasks}\left(contour\right)$  3:$pts_{inner}\leftarrow \mathrm{goodFeaturesToTrack}\left(I_{t-1},mask_{inner}\right)$  4:$pts_{outer}\leftarrow \mathrm{goodFeaturesToTrack}\left(I_{t-1},mask_{outer}\right)$  5:**if** $|pts_{inner}|\;<\tau_{pts}\mathrm{or}\left|pts_{outer}\right|<\tau_{pts}$ **then**  6:   **return** STATIC Not enough points for reliable judgment  7:**end if**  8:$flow_{inner}\leftarrow \mathrm{calcOpticalFlow}\left(I_{t-1},I_{t},pts_{inner}\right)$  9:$flow_{outer}\leftarrow \mathrm{calcOpticalFlow}\left(I_{t-1},I_{t},pts_{outer}\right)$10:$clusters\leftarrow \mathrm{DBSCAN}(pts_{inner},flow_{inner})$11:$cluster_{main}\leftarrow \mathrm{findLargestCluster}\left(clusters\right)$12:**if** $|cluster_{main}|/|pts_{inner}|\;<\tau_{ratio}$ **then**13:   **return** NON_RIGID {Chaotic motion}14:**end if**15:$motion_{main}\leftarrow \mathrm{calcMedianFlow}\left(cluster_{main}\right)$16:$motion_{bg}\leftarrow \mathrm{calcMedianFlow}\left(flow_{outer}\right)$17:**if** $distance\left(motion_{main},motion_{bg}\right)>\tau_{motion}$ **then**18:   **return** RIGID_MOTION {Global translation/rotation}19:**else**20:   $ratio_{outlier}\leftarrow \mathrm{calcOutlierRatio}\left(clusters,cluster_{main}\right)$21:   **if** $ratio_{outlier}>\tau_{outlier}$ **then**22:      **return** NON_RIGID Local motion detected23:   **else**24:      **return** STATIC Global stationary25:   **end if**26:**end if**

This fine-grained stage is selectively activated only for these ambiguous objects, ensuring that computational resources are used efficiently. It begins by computing a dense optical flow field within the object’s bounding box using RAFT [33] pretrained model (raft_things), which provides a complete, pixel-wise motion field. To robustly segment the locally moving parts, we model the distribution of these dense flow vectors using a Gaussian Mixture Model (GMM). The optical flow vectors (characterized by magnitude and direction) are first normalized to be invariant to lighting and motion speed. Then, the EM algorithm is employed to fit a two-component GMM (clustersNumber = 2) with a spherical covariance matrix type (EM::COV_MAT_SPHERICAL), set to terminate after 100 iterations or when the log-likelihood gain is less than 0.1. This process clusters the pixels into distinct motion patterns, allowing us to precisely segment the object’s moving components (e.g., the waving arm) from its static parts (e.g., the torso), enabling a more accurate exclusion of dynamic features from the SLAM process.

This paper assumes that the optical flows of both dynamic and static objects follow Gaussian distributions. The combination of optical flows from these two types of objects forms a sample set, which can be regarded as a linear combination of Gaussian distributions. By clustering the optical flow features using a Gaussian Mixture Model, the samples of optical flow features can be divided into multiple sets, each following an independent Gaussian distribution. The Gaussian Mixture Model is given by

(1) $$\begin{array}{c} P\left(X\right)=\sum_{i=1}^{k}\alpha_{i}N\left(X;\mu_{i};\sum_{i}\right) \end{array}$$

$\alpha_{i}$ is the mixing coefficient for each component and *N* represents the probability density function of the two-dimensional optical flow features:

(2) $$\begin{array}{c} P\left(X|\Phi \right)=\frac{1}{{2\pi |\sum |}^{1/2}}exp[-\frac{1}{2}{\left(X-\mu \right)}^{T}\sum^{-1}\left(X-\mu \right)] \end{array}$$

μ is the mean vector of each Gaussian component and ∑ is the covariance matrix of each Gaussian component.

To determine the category to which each optical flow feature belongs, the Expectation–Maximization (EM) algorithm is employed to perform maximum likelihood estimation of the parameters of the GMM. The EM algorithm typically consists of two steps:

E-step: First, given the current parameters, calculate the posterior probability $\Gamma (z_{ik})$ that each data point belongs to each Gaussian component. $z_{ik}$ represents the parameters of each sample *X*, and *k* corresponds to the *k*th Gaussian component in *X*.

(3) $$\begin{array}{c} \Gamma \left(z_{ik}\right)=\frac{\alpha_{k}P\left(X|\Theta \right)}{\sum_{j=1}^{k}\alpha_{j}P\left(X|\Theta \right)} \end{array}$$

M-step: Re-estimate the parameters based on the obtained posterior probabilities and update the model:

(4) $$\begin{array}{c} {\hat{\Theta }}_{MLE}=argmax\left(L\left(X|\Theta \right)\right) \end{array}$$

where L(X|Θ) is the log-likelihood function of the GMM. Repeat the EM algorithm until the change in the log-likelihood function is less than a set threshold or the maximum number of iterations is reached.

**Figure 6:**
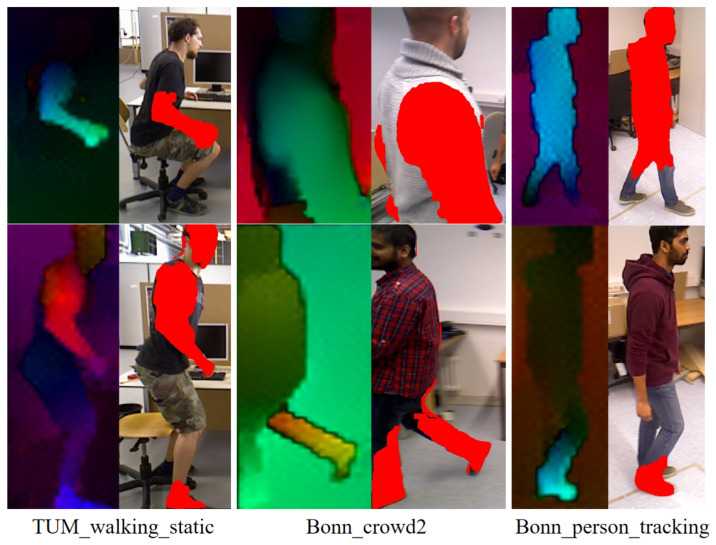
Fine-grained segmentation of dynamic targets. The left of each pair shows the optical flow visualization (brighter colors denote stronger motion), which guides the final segmentation mask (red overlay) shown on the right.

Figure 6 presents the visualization results of fine-grained segmentation for some scenes in the TUM and Bonn datasets [34]. Since the algorithm in this section calculates optical flow and makes motion judgments for individual bounding boxes, the size of each group of images varies based on the actual target, leading to inconsistencies in size as shown in the figure. For each group of images, the left side represents the optical flow visualization results based on combinations of color and brightness changes, where different colors represent different motion directions, and the brightness of the colors reflects the speed of motion. The right side of each group of images highlights the identification and segmentation results of local motion regions in the image. Our method effectively captures complex, non-rigid body movements (TUM_walking_static), partial subjects (Bonn_crowd2), and walking persons (Bonn_person_tracking), demonstrating its robustness and accuracy.

However, we acknowledge that the performance of our fine-grained segmentation is linked to the accuracy of the underlying dense optical flow estimation from RAFT. Potential failure cases arise in scenarios where the fundamental assumptions of optical flow are violated. For instance, on large, textureless surfaces (e.g., a monochromatic T-shirt), RAFT may struggle to produce reliable flow vectors, potentially leading to incomplete or hollow dynamic masks where moving regions are missed. Similarly, abrupt illumination changes or severe motion blur can violate the brightness constancy assumption, which may introduce noisy flow vectors and result in small, falsely identified dynamic regions. Recognizing these limitations is crucial for understanding the operational envelope of the proposed method.

By employing Fine-grained Segmentation based on dense optical flow clustering, we obtain a pixel-level identification of the object’s moving parts, which allows us to truly achieve the goal of detecting local object motion.

### 3.3. Three-Dimensional Gaussian Splatting

Maps serve as a bridge for mobile robots to interact with their environment. Accurate 3D reconstructed maps can help them better understand scenes and provide necessary information for subsequent tasks such as navigation and obstacle avoidance. Currently, the maps created by mainstream and mature visual SLAM systems are sparse and only used for robot localization. Traditional dense visual SLAM systems suffer from poor reconstruction quality and low detail clarity, while SLAM systems based on NeRF have long rendering times and do not meet real-time requirements. Therefore, this paper adopts a hybrid map representation based on geometric features and 3D Gaussians, combining ORB geometric features from visual SLAM with 3D Gaussians. Geometric feature points are used to estimate pose, while an efficient training strategy is designed to optimize the parameters of the 3D Gaussians.

The hybrid map consists of two types of map points: geometric feature points used for localization and 3D Gaussian training and 3D Gaussian points containing rich spatial information. The geometric feature points are primarily derived from the 3D map points in the geometric map generated by visual SLAM, inheriting the properties of ORB feature points and the characteristics of 3D Gaussians. Since SLAM systems excel in localization, the information of geometric feature points is mainly updated during the optimization process of the geometric map and is synchronously updated to the hybrid map. The 3D Gaussian points, which do not have ORB feature-related attributes, are mainly generated through densification algorithms and are used to fill in areas that cannot be covered by sparse geometric feature points. Three-dimansional Gaussians possess excellent scene representation capabilities, playing a crucial role in precise scene fitting and high-accuracy three-dimensional reconstruction.

The system is mainly divided into two threads: localization and mapping. Initially, camera pose estimation, including rotation R∈SO(3) and translation *t*, is performed through consecutive image frames. Subsequently, a sparse map composed of keyframes and map points is constructed by the geometric mapping thread. Based on the keyframes and their corresponding map points in the sparse geometric map, a hybrid map is incrementally created. The hybrid map employs explicit 3D Gaussians as the basic units for rendering, containing parameters such as center position μ, opacity α, 3D covariance matrix ∑, and color *c*. During the 3D Gaussian Splatting rendering process, the system projects 3D Gaussians in space onto a pixel-based image plane, sorts these Gaussian projections, and computes the value for each pixel.

Rendering a keyframe at a specific pose (R,t) involves calculating the contribution of all 3D Gaussian points in the hybrid map to the pixels. The 3D Gaussians in the hybrid map are projected onto the two-dimensional image plane, and the projected 2D Gaussians are sorted by depth values to ensure correct handling of occlusion relationships in subsequent rendering processes. The final color of each pixel is then calculated using the following alpha compositing formula:

(5) $$C\left(R,t\right)=\sum_{i\in N}c_{i}\alpha_{i}\prod_{j=1}^{i-1}\left(1-\alpha_{j}\right)$$

where *N* denotes the number of 3D Gaussians in the map, $c_{i}$ represents the color values output by spherical harmonics, and $\alpha_{i}$ denotes the opacity.

Due to the inability of the sparse geometric map from visual SLAM to meet the requirements of high-quality 3D reconstruction, this paper designs a 3D Gaussian densification algorithm to generate more 3D Gaussian points, capturing more detailed features and achieving a fine depiction of the scene. The original 3D GS method employs splitting and cloning techniques. In under-reconstructed areas where the current 3D Gaussians cannot accurately fit the necessary structures, resulting in blank regions, the algorithm simply creates Gaussian points of the same size and moves them along the direction of the positional gradient. For over-reconstructed areas, where a single 3D Gaussian has too broad a coverage to precisely outline complex shapes, the algorithm subdivides overly large 3D Gaussian points into smaller units.

However, the densification algorithm for 3D GS alone cannot provide sufficient parameters for the hybrid map, and many regional features remain difficult to capture. Additionally, splitting and cloning do not fully utilize accurate geometric feature information. Therefore, this chapter proposes a densification algorithm based on geometric features to further increase the information density of the map. Depending on the camera mode of the system, different strategies are adopted for monocular, stereo, and RGB-D modes. In RGB-D mode, the depth information of inactive two-dimensional feature points is directly utilized to project them into three-dimensional space. In monocular mode, the depth values of the closest active two-dimensional feature points are used to estimate the depths of these inactive points. In stereo mode, a stereo matching algorithm is employed to estimate the depths of inactive two-dimensional feature points. By utilizing geometric features from adjacent areas to densify the elements of the hybrid map, the system can significantly increase the density of feature points.

**Figure 7:**
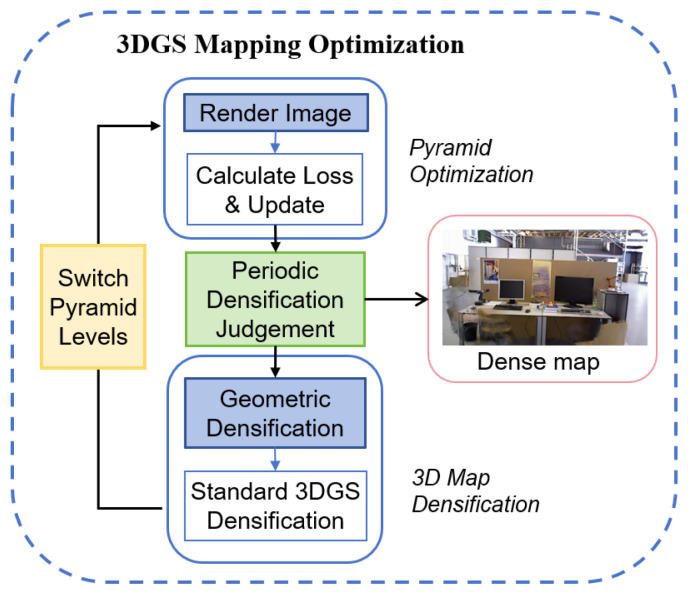
Workflow of the integrated pyramid optimization and densification process. The optimization iterates through pyramid levels, periodically triggering a dual densification step that combines our novel geometric densification with standard 3DGS techniques.

To clarify the interplay between our Gaussian pyramid-based optimization and the geometric feature-based densification, we present a detailed workflow in Figure 7. The process begins by initializing 3D Gaussians from the map feature points provided by the SLAM front-end. The optimization then iterates through the levels of a Gaussian pyramid, starting from the coarsest level to establish a robust global structure and progressively moving to finer levels to refine details. Crucially, within this optimization loop, our novel geometric densification and the standard 3DGS densification are periodically triggered. This ensures that new geometric details from the SLAM system are continually integrated and refined alongside the existing Gaussians, synergistically improving map quality and completeness.

The Gaussian Pyramid is a set of images that exhibit different levels of detail, constructed by continuously applying Gaussian smoothing and downsampling techniques to the original image. At the initial stage of training, the features from the top layer of the pyramid (the layer with the coarsest image representation) are used to guide the optimization of 3D Gaussian parameters. As the training iterations progress, on the one hand, the geometric parameters are densified to improve accuracy and detail representation, and on the other hand, the next layer of the pyramid is accessed to obtain new real image values. This process iterates repeatedly, gradually capturing and learning more detailed geometric features of the scene until reaching the bottom layer of the Gaussian Pyramid. The optimization process using a Gaussian Pyramid with layers can be expressed by the following formula:

(6) $$\left\{\begin{array}{cc} & t_{0}:argminL\left(I_{r}^{n},GP^{n}\left(I_{gt}\right)\right),\\ & t_{1}:argminL\left(I_{r}^{n-1},GP^{n-1}\left(I_{gt}\right)\right),\\ & \ldots \\ & t_{n}:argminL\left(I_{r}^{0},GP^{0}\left(I_{gt}\right)\right). \end{array}\right.$$

$GP^{n}\left(I_{gt}\right)$ denotes the real image in the *n*th level of the pyramid, and optimization is achieved by minimizing the photometric loss between the rendered image and the real image at each level of the pyramid.

For the rendered image and the real image, the loss is calculated as follows. Finally, parameters are updated through gradient backpropagation to achieve the optimization of 3D Gaussians.

(7) $$L=\left(1-\lambda \right)|I_{r}-I_{gt}{|}_{1}+\lambda \left(1-SSIM\left(I_{r},I_{gt}\right)\right)$$

The loss is measured by the $L_{1}$ and D-SSIM loss between the rendered image $I_{r}$ and the real image $I_{gt}$.

**Figure 8:**
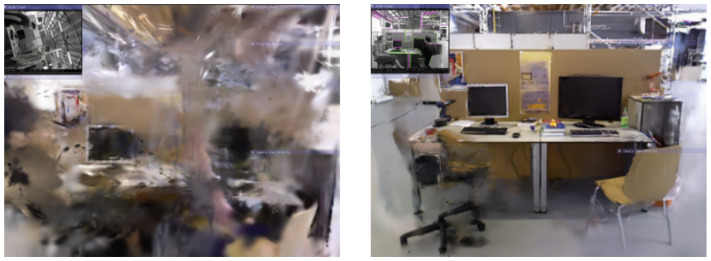
The left image shows the original rendering result based on 3D GS, while the right image shows the result after excluding the influence of dynamic objects in this paper.

This paper combines 3D scene representation with Gaussian Pyramid training and geometric densification algorithms, aiming to significantly improve training efficiency while ensuring that 3D Gaussians can accurately capture the detailed features of the scene. Based on the dynamic object segmentation module of our system, we successfully achieved the identification and segmentation of dynamic objects, which is crucial for ensuring that the feature points belonging to dynamic objects do not interfere with the map reconstruction process. By effectively excluding these interfering feature points, our 3D GS model is able to produce a clearer and less noisy map reconstruction, as shown in Figure 8.

To better position our contribution within the landscape of existing research, we summarize the primary methodologies for dynamic SLAM in Table 1. While prior approaches based on multi-view geometry, semantic priors, or dense optical flow have their respective strengths, they also suffer from significant drawbacks, such as failure in dynamic-cluttered scenes, inability to handle unknown objects, or prohibitive computational costs. Our proposed coarse-fine cascaded strategy is designed to synergistically combine the advantages of these methods while mitigating their limitations. By using an efficient sparse optical flow check to filter the majority of objects, we achieve real-time performance, and by reserving a fine-grained dense flow analysis for only the most complex cases, we retain the ability to accurately segment both global and local movements, thereby enhancing both localization robustness and mapping quality.

## 4. Experimental Results and Discussion

The algorithm proposed in this paper is combined with the visual ORB-SLAM framework to conduct experimental tests focusing on positioning and 3D reconstruction accuracy. The experimental test scenarios include static, high-dynamic, and low-dynamic environments, divided into three groups of experiments. The first group is an experiment on the pose optimization algorithm based on dynamic object segmentation, comparing it with ORB-SLAM and advanced SLAM methods in low-dynamic and high-dynamic scenarios. The second group of experiments is an ablation study comparing overall judgment and fine-grained segmentation methods, validating the improvement effect of combining these two modules on overall pose optimization in dynamic environments. The third group of experiments focuses on the effectiveness of 3D reconstruction based on geometric features and 3D Gaussians. All experiments were conducted on an Ubuntu 20.04 operating system equipped with an Intel r9 CPU, RTX3060 GPU, and 16 GB of RAM.

### 4.1. Computational Cost Analysis

A key motivation behind our coarse-fine dynamic segmentation pipeline is to significantly reduce the computational overhead associated with dense optical flow methods. To quantify this improvement, we conducted a performance analysis on the TUM fr3_walking_static sequence. We compared the average processing time of our proposed method against a baseline approach that employs dense optical flow (RAFT) for all initial motion judgments.

The results are presented in Table 2. The baseline method (‘Dense Flow Only’) reflects the performance of the original algorithm, which computes dense optical flow for every potential dynamic object in each frame. In contrast, our coarse-fine cascaded method first employs a highly efficient sparse flow and DBSCAN clustering stage for preliminary classification. The computationally intensive dense flow analysis is only invoked for objects identified as having complex local motion.

The experimental data demonstrate the substantial efficiency gains of our approach. By leveraging sparse optical flow for initial screening, our method reduces the average processing time per object from 474.98 ms to just 39.18 ms, achieving a 91.7% improvement in speed. More critically, our cascaded framework successfully avoids unnecessary dense flow computations in **88.6%** of cases. This dramatic reduction in computational load is crucial for maintaining the real-time performance of the SLAM system in dynamic environments, validating the effectiveness of our hierarchical design. In terms of overall system throughput, this translates to an increase in processing frame rate from approximately 0.6 FPS with the dense-only approach to 21 FPS with our method on the test hardware.

### 4.2. Localization Evaluation

#### 4.2.1. Experiments on the TUM Dataset

As shown in Table 3, static methods like MonoGS [30] and Splat-SLAM [35] struggle in dynamic environments, with MonoGS failing catastrophically (e.g., 44.2 cm ATE RMSE on f3/walking_half). In contrast, our proposed method maintains high accuracy across all sequences, achieving a superior average ATE RMSE of 2.02 cm. This quantitatively validates that our motion analysis is critical for robust 3DGS-SLAM in dynamic scenes.

For testing our algorithm, we chose five sequences from TUM RGB-D dataset and used Absolute Trajectory Error (ATE) and Relative Pose Error (RPE) as metrics to assess the accuracy of our system. The comparative results of our method against ORB-SLAM3, DS-SLAM, and SG-SLAM are presented in Table 4 and Table 5. Despite the presence of highly dynamic objects in these scenarios, our proposed algorithm demonstrates significant improvements over ORB-SLAM3, achieving an accuracy rate of up to 95.4%. It also yields results comparable to some dynamic SLAM methods, with typical errors ranging from 1 to 3 cm. Notably, the standard deviation (STD) of the error is also significantly reduced across most sequences. This underscores the enhanced stability and consistency of our method, indicating improvements not only in average accuracy but also in tracking robustness.

**Figure 9:**
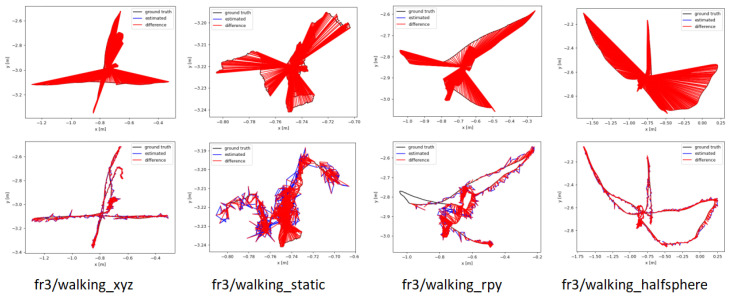
Estimated camera trajectory results of ORB-SLAM3 (**top**) and the method proposed in this paper (**bottom**) on sequences from the TUM dataset, along with the differences from the ground truth values.

**Figure 10:**
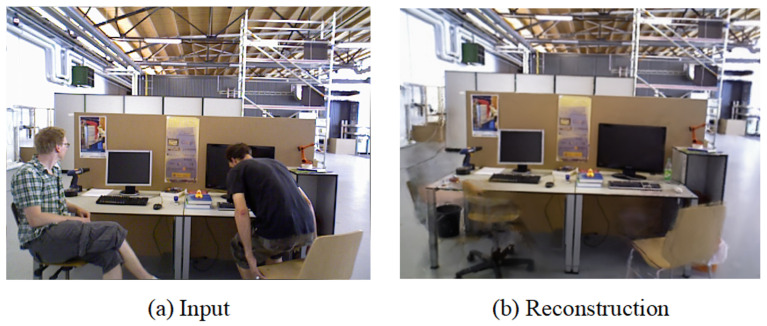
Rendering results of static background in fr3/walking_halfsphere for the TUM dataset using the method in this paper.

Figure 9 shows the ATE between the algorithm proposed in this paper, ORB-SLAM3, and the ground truth trajectory, with trajectory errors marked in red. The experimental results indicate that ORB-SLAM3 exhibits larger errors in high-motion sequences, whereas our proposed method can estimate a trajectory that is closer to the original path. Additionally, Figure 10 displays the 3D GS rendering results for a sequence. By excluding the influence of feature points on dynamic objects, our system focuses more on rendering the static parts and can automatically fill in missing parts from previous renderings based on other frames to repair the background. This capability allows our system to complete the rendering of static scenes effectively.

#### 4.2.2. Experiments on the Bonn Dataset

The Bonn RGB-D dataset comprises 24 sequences featuring dynamic objects, and we chose four representative highly dynamic sequences for performance evaluation experiments.

To further challenge our system, we conducted evaluations on the more demanding Bonn RGB-D dataset, which features sequences with highly complex and dominant dynamic objects. Table 6 presents the comparative ATE RMSE results of our method against several state-of-the-art dynamic SLAM systems, including DynaSLAM, SG-SLAM, and DN-SLAM [36], as well as the baseline ORB-SLAM3. Despite the increased complexity of dynamic information in the Bonn sequences compared to the TUM dataset, our method remains effective in removing dynamic regions and achieving highly competitive results. Even when dynamic regions dominate the scene, such as in the crowd sequences, our method can still adjust to produce accurate localization outcomes, significantly outperforming the ORB-SLAM3 baseline. However, a closer analysis reveals nuanced performance variations. Notably, on highly dynamic sequences such as crowd and crowd2, our method, while still robust, exhibits slightly higher errors compared to its performance on sequences with fewer moving objects (e.g., person_tracking). We attribute this primarily to two interconnected challenges inherent in dense crowd scenarios: First, the prevalence of severe and frequent occlusions complicates the multi-object tracking process. Although ByteTrack is robust, sustained occlusions can lead to temporary tracking failures. When a track is lost, the lack of a continuous two-frame correspondence prevents our optical flow-based motion analysis, forcing the system to temporarily rely on potentially dynamic features. Second, the motion patterns in dense crowds are often chaotic and tightly coupled. When multiple people move in close proximity, the annular regions used for our coarse sparse-flow analysis can overlap, mixing motion cues from different individuals. While our DBSCAN clustering is designed to handle this, in extreme cases, it can lead to a suboptimal estimation of the dominant motion, resulting in minor inaccuracies in the final pose estimation. Future work could explore integrating more sophisticated instance-aware motion models to further disentangle complex, multi-body movements in such challenging scenarios.

**Figure 11:**
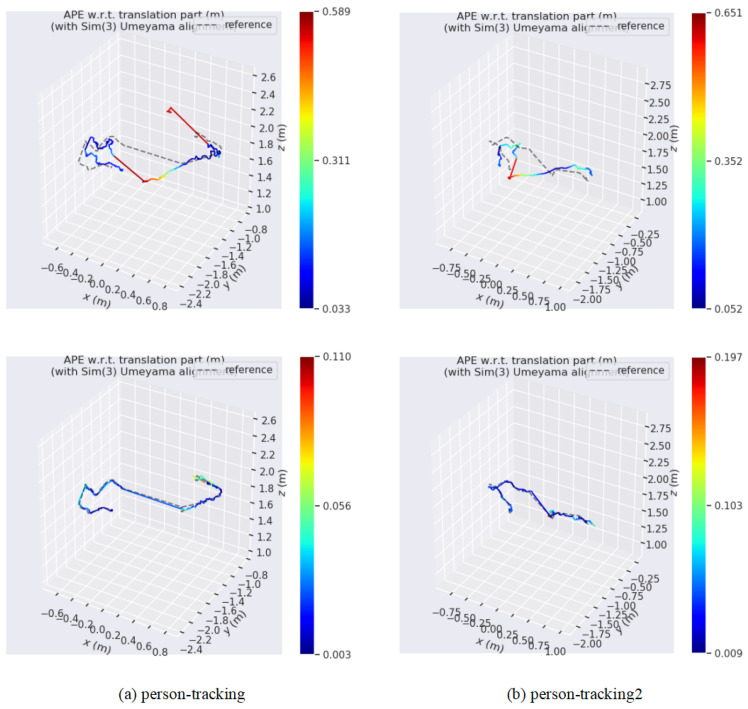
Estimated camera trajectory results of ORB-SLAM3 (**top**) and the method proposed (**bottom**) on the person-tracking sequences of the Bonn dataset, along with the differences from the ground truth values.

**Figure 12:**
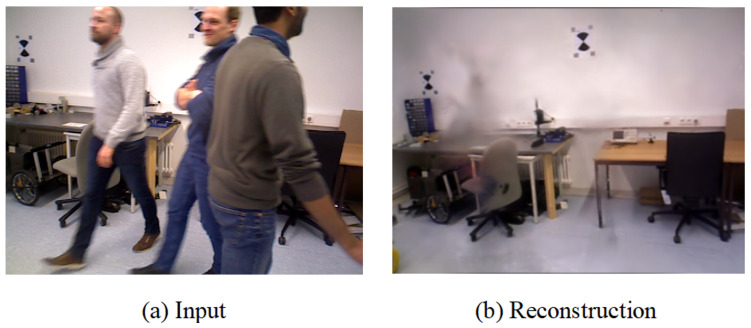
Three-dimensional reconstruction results of the rgbd_bonn_crowd sequence from the Bonn dataset using the method proposed in this paper.

Figure 11 showcases the differences between the estimated trajectories obtained by various algorithms in human tracking experiments on Bonn sequences and the corresponding ground truth trajectories. ORB-SLAM3 exhibits significant estimation errors in highly dynamic scenes, primarily due to the influence of dynamic objects. However, our method addresses this issue effectively, resulting in more precise outcomes. Additionally, Figure 12 displays the reconstruction results for one of these sequences, where, based on accurate segmentation of dynamic object regions, the map rendering excludes these areas and constructs a complete static background.

#### 4.2.3. Ablation Experiments

To further illustrate the necessity of our cascaded coarse-fine approach, we provide a qualitative analysis of a challenging scenario from the TUM. As shown in our ablation study (Table 7), both the coarse-only (OURS(C)) and fine-only (OURS(F)) methods yield suboptimal results compared to our full pipeline (OURS(C+F)).

**Figure 13:**
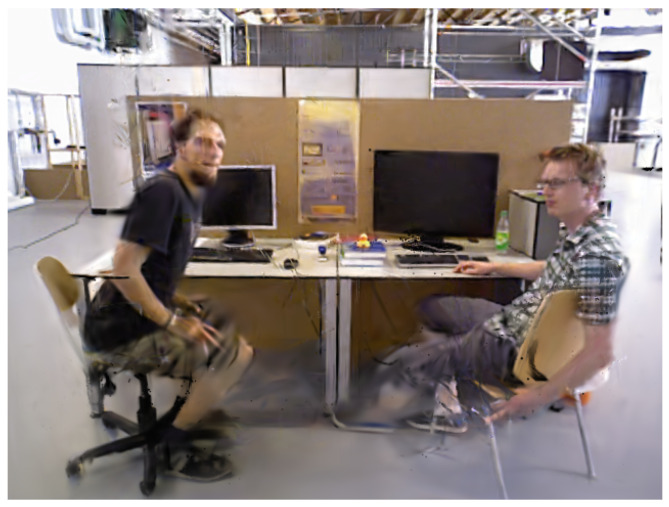
Failure case of the fine-grained-only segmentation on the TUM sequence. Without the coarse-stage prescreening, the dense flow clustering is sensitive to noise, leading to the erroneous removal of static parts of the person, such as the feet.

The coarse-only method, relying on averaged sparse flow, fails to detect subtle non-rigid arm movements against a dominant static torso, incorrectly classifying the entire person as static. This allows dynamic features to corrupt the tracking process. More revealingly, the fine-only method (OURS(F)) also shows a significant degradation in accuracy. Without the coarse-stage prescreening, the GMM clustering is applied directly to the raw dense optical flow field of every potential dynamic object. We found that GMM clustering is sensitive to outliers and noise inherent in dense flow estimation, especially in textureless or reflective regions. As visualized in Figure 13, without the robust initial classification provided by our coarse stage, the GMM can misinterpret noisy flow vectors as a distinct motion cluster. This leads to the erroneous segmentation of static parts of the object (e.g., parts of the torso or legs) as dynamic, unnecessarily removing valid features for tracking, and resulting in compromised pose estimation.

Our full coarse-fine method resolves both failure modes. The coarse stage reliably handles clear static/dynamic cases, while also acting as a crucial filter that passes only truly ambiguous, non-rigid motion cases to the fine-grained stage. This ensures that the GMM is applied only when necessary and to cleaner, more well-defined motion problems, guaranteeing both high accuracy and efficiency.

### 4.3. Rendering Quality Evaluation

**Figure 14:**
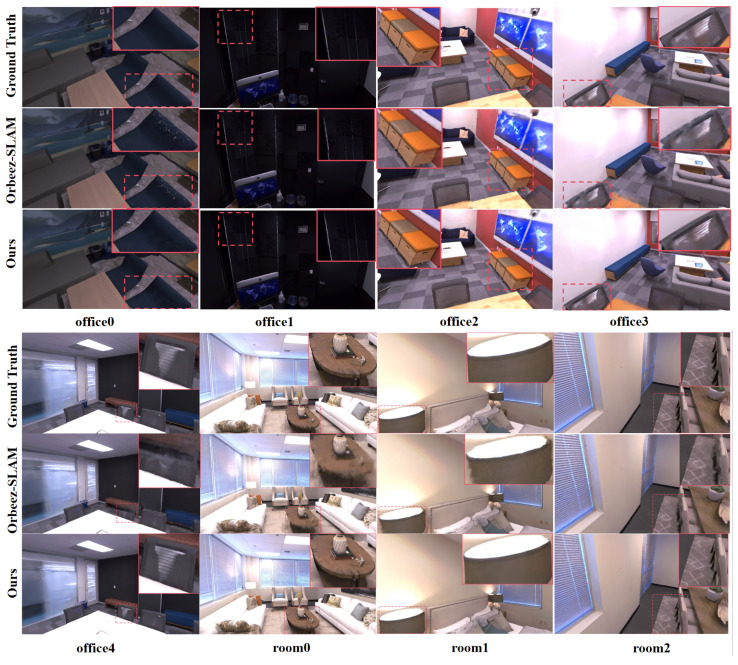
A visual comparison of the rendering results between Orbeez-SLAM and the method proposed in this paper on the Replica dataset.

We evaluated the rendering quality on eight sequences from the Replica RGB-D dataset and primarily compared the visual results with the Orbeez-SLAM method, which also integrates ORB-SLAM. As illustrated in Figure 14, our method excels in rendering quality in certain details, leading to the production of higher-quality map models during the final mapping process.

To further assess the quality of image rendering, we employed Peak Signal-to-Noise Ratio (PSNR↑), Structure Similarity Index Measure (SSIM↑), and Learned Perceptual Image Patch Similarity (LPIPS↓) as our evaluation metrics. According to the experimental data in Table 8, we compared our method with several advanced NeRF-based SLAM methods [20,37]. The 3D reconstruction method presented in this paper demonstrates certain advantages in terms of image reconstruction quality, similarity measurement, and visual effects. In particular, when compared with Orbeez-SLAM, which also integrates ORB-SLAM and neural radiance fields, the 3D Gaussian model and optimization strategy adopted in this paper yield better results, with PSNR improved by 1.88 dB, SSIM increased by 0.026, and LPIPS decreased by 0.041, indicating a reduction in perceptual differences between images.

### 4.4. Discussion and Limitations

Our experimental results confirm the robustness and accuracy of our proposed coarse-fine dynamic SLAM framework. The modular design facilitates easy integration with other SLAM systems and supports future upgrades, such as replacing the instance segmentation network with zero-shot models for improved generalization. This scalability and adaptability make the system highly suitable for robotics applications in dynamic environments, offering a solid platform for further development in real-world settings. However, we identify two primary areas for future improvement.

First, as observed in the Bonn dataset (Table 6), localization errors are slightly elevated in dense crowd scenes. This is mainly attributed to frequent, severe occlusions that can challenge the multi-object tracker, and chaotic, coupled motions that occasionally lead to suboptimal motion clustering.

Second, while our method produces clean reconstructions of the static background, residual visual artifacts can appear in the 3DGS map under intense dynamic activity. These include slight blurring or “ghosting” in frequently occluded areas and minor “floating” artifacts from transient segmentation errors.

Future work will focus on addressing these challenges. We plan to integrate more sophisticated, instance-aware motion models to better handle tightly coupled crowd movements. Furthermore, we will explore uncertainty-aware rendering and stronger temporal consistency constraints in the mapping backend to mitigate visual artifacts and further enhance reconstruction quality. Additionally, we plan to investigate the integration of advanced reinforcement learning algorithms to improve the agent’s trajectory planning and interaction with dynamic elements. The comparative evaluation of DDPG variants for trajectory tracking by Ben Hazem et al. [38] suggests that methods like TD3-ADX could offer superior precision and faster convergence, providing a valuable research direction for future iterations of our system.

## 5. Conclusions

This paper introduces a robust 3DGS-SLAM system designed for effective operation in dynamic environments. A novel, cascaded coarse-to-fine motion segmentation framework is proposed, combining the efficiency of sparse optical flow with the precision of dense optical flow clustering. This approach enables accurate identification and filtering of both global and local dynamic elements, overcoming a critical challenge that limits prior static 3DGS-SLAM methods.

The method achieves state-of-the-art localization accuracy, reducing the ATE RMSE by up to 95.4% compared to ORB-SLAM3 on dynamic sequences while significantly reducing motion analysis time by 91.7%. The system also demonstrates superior mapping quality, outperforming neural SLAM baselines like Orbeez-SLAM, with an average LPIPS reduction of 0.041 on the static Replica dataset. The proposed framework provides a practical solution for dynamic SLAM, addressing the limitations of existing paradigms by combining the efficiency of traditional SLAM with advanced motion segmentation techniques. Sparse and dense optical flow are used to efficiently filter dynamic elements, ensuring real-time performance while maintaining the efficiency of classic SLAM for static scenes. 

## Figures and Tables

**Table 1:** Comparison of different dynamic SLAM methodologies.

Methodology	Core Principle	Advantages	Disadvantages
**Multi-view Geometry**	Identifies motion outliers based on geometric model inconsistencies (e.g., epipolar constraints).	No prior knowledge or training required.	Fails when dynamic objects dominate the scene. Sensitive to camera motion (e.g., pure rotation).
**Semantic Priors**	Uses object detection or semantic segmentation to pre-identify potentially dynamic object categories.	Can handle unknown motion patterns. Semantically meaningful map.	Fails for unknown dynamic object categories. Risks removing static features on dynamic-class objects.
**Optical Flow (Dense)**	Computes per-pixel motion vectors and segments regions with distinct motion patterns.	Provides fine-grained motion details. Independent of object category.	Prohibitively high computational cost for real-time use. Sensitive to illumination changes and textureless regions.
**OURS (Coarse-Fine)**	**Cascaded approach: Uses efficient sparse optical flow for coarse screening, and invokes dense flow with clustering only for ambiguous, non-rigid targets.**	**Efficient and Robust:** Achieves high accuracy by combining sparse and dense flow while drastically reducing computational load. **Accurate:** Preserves static features on dynamic objects while segmenting fine-grained local motion.	Performance relies on the quality of the underlying optical flow estimation. Introduces additional parameters for the cascaded logic.

**Table 2:** Computational cost analysis on TUM fr3_walking_static.

Method	Avg. Time/Object (ms)	Dense Flow Calls (%)
Dense Flow Only (Baseline)	474.98	100%
Ours (Cascaded Sparse+Dense)	**39.18**	**11.4%**
Ours (Sparse Flow Only)	11.34	-
**Improvement**	**91.7%**	**88.6% Reduction**

**Table 3:** Comparison of Absolute Trace Error (cm) on dynamic sequences from the TUM RGB-D dataset. We explicitly compare our method against prior 3DGS-SLAM works designed for static scenes. Best results are in bold.

Method	f3/ws	f3/wxyz	f3/wr	f3/wh	Avg.
MonoGS	1.34	21.5	17.4	44.2	21.11
Splat-SLAM	2.37	1.86	3.94	3.38	2.89
**OURS**	**0.87**	**1.38**	**3.08**	**2.75**	**2.02**

**Table 4:** Comparison of Absolute Trace Error (M) results of different methods on TUM. Best results are bold.

Sequences	ORB-SLAM3		DS-SLAM		SG-SLAM		OURS		Improvements	
	RMSE	STD	RMSE	STD	RMSE	STD	RMSE	STD	RMSE	STD
fr3/walking_xyz	0.3021	0.1402	0.0247	0.0161	0.0152	0.0075	**0.0138**	**0.0066**	95.4%	95.2%
fr3/walking_static	0.0256	0.0144	0.0081	0.0036	**0.0073**	**0.0034**	0.0087	0.0035	66.0%	75.6%
fr3/walking_rpy	0.1824	0.1033	0.4442	0.2350	0.0324	0.018	**0.0308**	**0.0179**	82.6%	82.2%
fr3/walking_half	0.4845	0.2648	0.0303	0.0159	**0.0268**	0.0134	0.0275	**0.0130**	94.3%	95.0%
fr3/sitting_static	0.0380	0.0178	0.0065	0.0033	**0.0060**	**0.0029**	0.0064	0.0032	83.1%	82.0%

**Table 5:** Comparison of Relative Pose Error (M/S) results of different methods on TUM. Best results are bold.

Sequences	ORB-SLAM3		DS-SLAM		SG-SLAM		OURS		Improvements	
	RMSE	STD	RMSE	STD	RMSE	STD	RMSE	STD	RMSE	STD
fr3/walking_xyz	0.2288	0.0947	0.0333	0.0229	0.0194	0.0100	**0.0180**	**0.0084**	92.1%	91.1%
fr3/walking_static	0.0136	0.0066	0.0102	0.0048	**0.0100**	0.0051	0.0107	**0.0045**	21.3%	31.8%
fr3/walking_rpy	0.1262	0.0873	0.1503	0.1168	0.0450	0.0262	**0.0421**	**0.0238**	66.6%	72.7%
fr3/walking_half	0.2578	0.1299	0.0297	0.0152	0.0279	0.0146	**0.0264**	**0.0119**	89.7%	90.8%
fr3/sitting_static	0.0142	0.0083	0.0078	0.0038	**0.0075**	**0.0035**	0.0083	0.0041	41.5%	50.6%

**Table 6:** Comparison of Absolute Trace Error (M) results of different methods on Bonn. Best results are bold.

Sequences	ORB-SLAM3		DynaSLAM		SG-SLAM		DN-SLAM		OURS	
	RMSE	STD	RMSE	STD	RMSE	STD	RMSE	STD	RMSE	STD
crowd	1.3297	0.4830	0.025	0.013	**0.0234**	**0.0143**	0.025	0.016	0.0269	0.0176
crowd2	1.3176	0.5926	0.029	0.017	0.0584	0.0406	**0.028**	**0.017**	0.0292	0.0181
crowd3	0.9593	0.3638	0.037	0.021	0.0319	0.0219	0.026	0.014	**0.0259**	**0.0142**
person_tracking	0.6623	0.3089	0.047	0.015	0.0400	0.0139	0.038	0.015	**0.0353**	**0.0122**
person_tracking2	0.9478	0.4512	0.126	0.072	**0.0376**	0.0154	0.042	0.017	0.0379	**0.0141**
balloon	0.0599	0.0253	0.034	0.015	-	-	0.030	**0.012**	**0.0285**	0.0130
balloon2	0.2867	0.1818	0.032	0.014	-	-	**0.025**	**0.012**	0.0278	0.0156
synchronous	1.1267	0.5569	-	-	0.3229	0.1824	-	-	**0.0388**	**0.0325**
synchronous2	1.0474	0.4320	-	-	0.0164	0.0126	-	-	**0.0089**	**0.0055**

**Table 7:** Comparison of Root Mean Square Error (RMSE[M]) results of our methods. Best results are bold.

Sequences	OURS(C)		OURS(F)		OURS(C+F)	
	RMSE	STD	RMSE	STD	RMSE	STD
fr3/walking_xyz	0.0162	0.0081	0.1116	0.0683	**0.0155**	**0.0073**
fr3/walking_static	0.0118	0.0055	0.1841	0.0965	**0.0089**	**0.0049**
fr3/walking_rpy	0.0411	0.0259	0.3639	0.2656	**0.0308**	**0.0179**
fr3/walking_half	0.0358	0.0178	0.1687	0.1296	**0.0275**	**0.0134**
fr3/sitting_static	0.0082	0.0041	0.0074	0.0038	**0.0066**	**0.0031**

**Table 8:** Rendering performance comparison Of RGB-D SLAM methods on Replica.

Sequences	Metric	Of0	Of1	Of2	Of3	Of4	R0	R1	R2
NICE-SLAM	PSNR ↑	29.07	30.34	19.66	22.23	24.94	22.12	22.47	24.52
	SSIM ↑	0.874	0.886	0.797	0.801	0.856	0.689	0.757	0.814
	LPIPS ↓	0.229	0.181	0.235	0.209	0.198	0.330	0.271	0.208
CoSLAM	PSNR ↑	34.14	34.87	28.43	28.76	30.91	27.27	28.45	29.06
	SSIM ↑	**0.961**	**0.969**	0.938	0.941	**0.955**	**0.910**	0.909	0.932
	LPIPS ↓	0.209	0.196	0.258	0.229	0.236	0.324	0.294	0.266
ESLAM	PSNR ↑	33.71	30.20	28.09	28.77	29.71	25.32	27.77	29.08
	SSIM ↑	0.960	0.923	**0.943**	**0.948**	0.945	0.875	0.902	0.932
	LPIPS ↓	0.184	0.228	0.241	0.196	0.204	0.313	0.298	0.248
Orbeez-SLAM	PSNR ↑	33.99	33.89	29.02	30.74	30.44	28.88	**30.92**	**32.69**
	SSIM ↑	0.935	0.815	0.919	0.919	0.932	0.881	**0.928**	**0.950**
	LPIPS ↓	0.086	0.096	0.132	0.118	0.146	0.183	0.130	0.096
OURS	PSNR ↑	**36.76**	**37.76**	**31.44**	**32.52**	**33.09**	**29.69**	29.38	31.52
	SSIM ↑	0.955	0.954	0.926	0.926	0.935	0.876	0.883	0.925
	LPIPS ↓	**0.070**	**0.061**	**0.095**	**0.085**	**0.079**	**0.109**	**0.098**	**0.068**

## Data Availability

The original contributions presented in this study are included in the article. Further inquiries can be directed to the corresponding author.
